# Acute Sarcopenia after Elective and Emergency Surgery

**DOI:** 10.14336/AD.2022.0404

**Published:** 2022-12-01

**Authors:** Alvin Shrestha, Melanie Dani, Paul Kemp, Michael Fertleman

**Affiliations:** ^1^Cutrale Perioperative and Ageing group, Imperial College London, London SW7 2BX, United Kingdom; ^2^National Lung and Health Institute, Imperial College London, London SW7 2BX, United Kingdom

**Keywords:** acute sarcopenia, muscle wasting, surgery, biomarkers

## Abstract

Sarcopenia is an increasingly recognised condition of loss of muscle mass and function. The European Working Group on Sarcopenia in Older People 2 (EWSOP2) updated their definition in 2018, emphasising the importance of low muscle strength in diagnosis. Acute sarcopenia has been arbitrarily defined as sarcopenia lasting less than 6 months. This review highlights the pathophysiology involved in muscle wasting following surgery, focussing on hormonal factors, inflammation, microRNAs, and oxidative stress. Biomarkers such as GDF-15, IGF-1 and various microRNAs may predict post-surgical muscle loss. The impact of existing sarcopenia on various types of surgery and incident muscle wasting following surgery is also described. The gaps in research found include the need for longitudinal studies looking in changes in muscle strength and quantity following surgery. Further work is needed to examine if biomarkers are replicated in other surgery to consolidate existing theories on the pathophysiology of muscle wasting

Sarcopenia is an increasingly recognised and researched geriatric syndrome of age-associated loss of muscle mass and function, linked with adverse outcomes such as falls, reduced quality of life, frailty and mortality [1]. Associated with a high economic burden, annual costs ascribed to sarcopenia have been estimated to be over $18 billion in the United States and £2.5 billion in the United Kingdom [2]. In 2018, the European Working Group on Sarcopenia in Older People (EWGSOP) updated their preceding 2010 definition: an individual has probable sarcopenia when low muscle strength is detected, and sarcopenia is confirmed by the addition of low muscle quantity/quality. When the triad of low muscle strength, low muscle quantity and low physical performance are all present, sarcopenia is considered severe.

Loss of muscle mass has been a well-established consequence of critical illness, so called intensive care unit acquired weakness (ICUAW), and may represent a disorder in the same spectrum as sarcopenia [3]. Frailty is also a geriatric syndrome related, but distinct, from sarcopenia, and may arise as a consequence. It is defined as a state of vulnerability to stressor events, resulting in poor resolution of homeostasis [4]. Freid’s frailty phenotype defines this as weight loss, self-reported exhaustion, low physical activity, weakness, and slow walking speed [4]. When considering that the latter two points overlap with the EWGSOP definition, the relationship between frailty and sarcopenia becomes easier to appreciate.

Sarcopenia which has lasted less than 6 months may be referred to as “acute” and is usually the result of an acute illness. Chronic sarcopenia refers to that persisting beyond 6 months, and may be related to progressive chronic conditions [5]. The prevalence of sarcopenia has been found to be 1-29% for older adults in the community and 10% for those in acute hospital care [6]. Studies are lacking in describing new, incident sarcopenia following acute illnesses, and particularly surgery, however. This review aims to explore the existing research on sarcopenia in surgical groups, its pathophysiology and how this may be affected by surgery.

## Diagnosing sarcopenia

Low muscle strength can be detected by measuring grip strength with a handheld dynamometer, with a cut-off for sarcopenia of <27 kg for men and <16 kg in women [7]. Due to its ease and excellent reproducibility, this technique has been advised by experts. An alternative method is the “chair stand/rise test”, whereby a time of greater than 15 seconds required for a participant to rise five times from a seated position represents the cut-off for sarcopenia [5].

Low muscle quantity can be confirmed non-invasively with cross-sectional assessment of selected muscle groups by magnetic resonance imaging (MRI) or computed tomography (CT), which are considered the gold standard. Specifically, mid-thigh muscle area has been shown to be strongly correlated with total body muscle volume [8]. In addition, cross-sectional area of muscles at the anatomical landmark of L3 vertebra has also been shown to correlate with whole body muscle, albeit to a weaker extent than mid-thigh [5]. Nevertheless, muscle evaluation at the L3 level has been opportunistically used in surgical patients where CT scans of pelvis are common - namely in cancers (where the imaging is performed for initial staging, then following treatment or for surveillance) and diseases of the gastrointestinal tract. Whilst imaging allows for serial monitoring of change in muscle size, exact measurement cut-offs to denote sarcopenia are currently undefined. In fact, due to the cost of and lack of access in primary care in obtaining CT or MRI, it is the dual-energy X-ray absorptiometry (DEXA) scan that has been recommended for determining total muscle quantity. DEXA scans which report an appendicular skeletal muscle mass (ASMM) per height of < 7.0 kg/m^2^ in men, and < 5.5 kg/m^2^ in women are defined as confirmed sarcopenia [5]. Muscle mass may also be estimated by bioelectrical impedance analysis (BIA), using a device to measure electrical conductivity; cut-off values are population-specific [5]. Muscle quality can be considered histologically as a shift from type II fibres to type I and fat infiltration [4,9]. As muscle biopsies cannot be routinely used in clinical practice for this purpose, imaging has been considered as a possible gateway to evaluate for quality. Both CT and MRI are able to determine fatty infiltration [10], while ultrasound echogenicity has shown promise but needs further evaluation [11]. However, no consensus has been made as to how imaging results can be quantified into determining quality as yet [5].

The EWGSOP2 recommends using a gait speed of ≤0.8 m/s (i.e., taking 5 seconds or longer to walk 4 m) as evidence for low physical performance. Other assessments include a composite Short Physical Performance Battery (SPPB) score of ≥8 and Timed Up and Go test (TUG; time taken to rise from seating walk 3 m, turn, return to the chair, and sit back down) of ≥20 s to establish severe sarcopenia.

### Pathophysiology of sarcopenia

**Figure 1.**
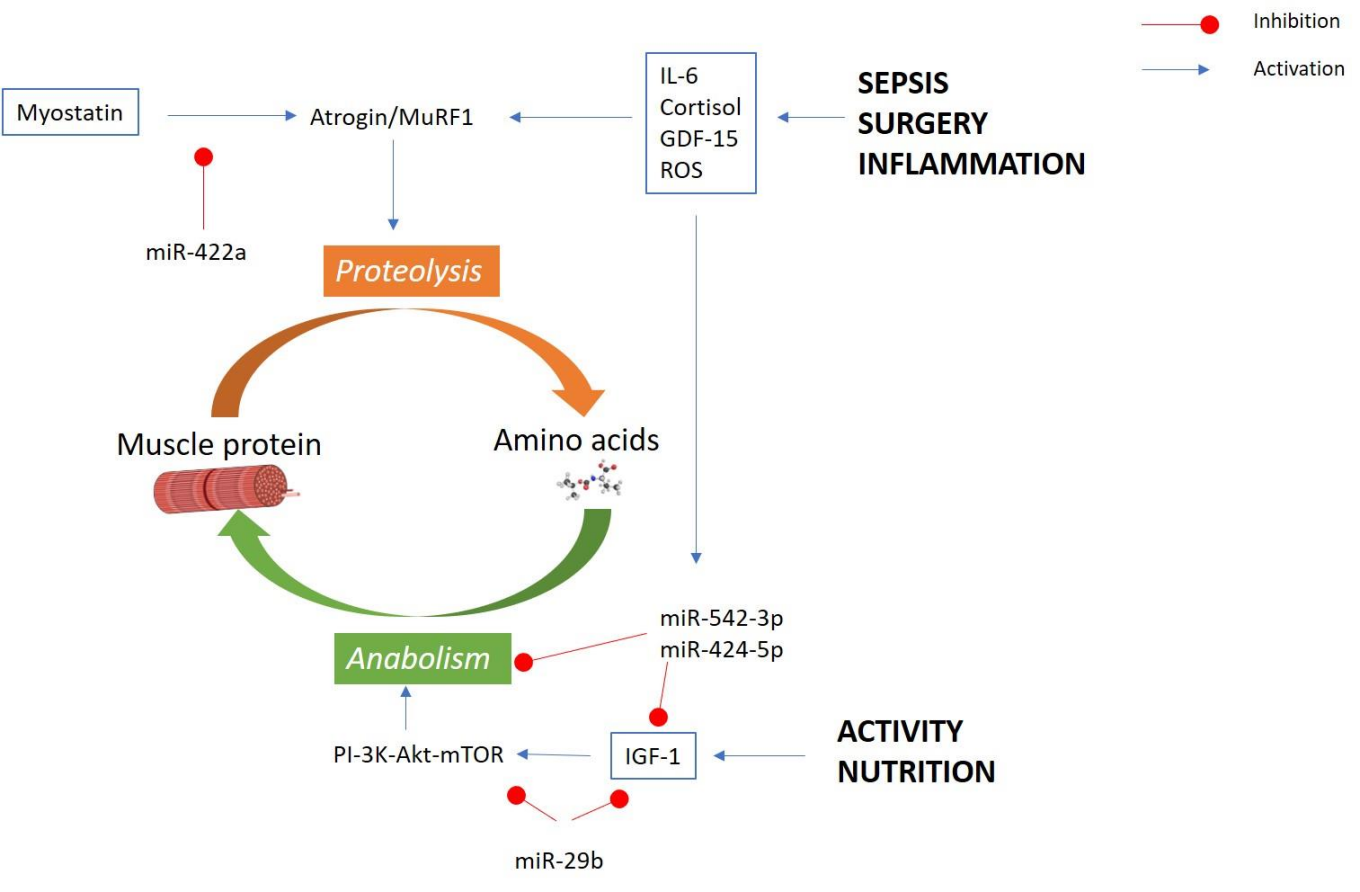
schematic demonstrating the key components in sarcopenia pathophysiology. The factors and pathways involved in the balance between proteolysis (leading to sarcopenia) and anabolism is shown. Blue line indicates activation of a pathway and red line indicates inhibition of a pathway.

Various pathways interact and contribute to skeletal muscle health and developing sarcopenia as portrayed in Figure 1. A net loss of muscle protein occurs when protein breakdown, proteolysis, exceeds synthesis, anabolism. Muscle protein is constantly turned over with a net synthesis of approximately 1-2% per day in healthy adult humans, balanced by an equivalent rate of protein breakdown such that total muscle mass is maintained [12]. Sarcopenia results from an imbalance in these rates with an excess of protein breakdown (proteolysis) leading to a net loss of muscle protein. The imbalance can arise from either a reduction in protein synthesis or an increase in protein breakdown that is greater than any change in the opposite direction and very small changes can lead to significant muscle loss over time [12,13]. Growth factors, including insulin and IGF-1, are known to stimulate protein synthesis and increase muscle mass [14]. These factors bind to the insulin and IGF-1 receptors and signal through the PI-3K-Akt-mTOR pathway to increase protein synthesis by activating translational initiation by phosphorylating a range of ribosomal targets including S6 kinase and 4E-binding protein 1. The best studied catabolic pathway in muscle is the ubiquitin ligase pathway in which the E3 ligase MuRF1 and Atrogin promote targeting of muscle proteins to the proteosome. This expression of these genes is increased by a wide range of factors including interleukins (e.g. IL-6), stress hormones (e.g. cortisol) and TGF-β family members (e.g. myostatin) [14]. Consistent with these suggestions, myostatin promotes muscle loss in mice [15] and has been associated with muscle mass in chronic disease [12], cortisol excess promotes muscle atrophy and both myostatin and cortisol have been shown to increase with age [16,17]. Similarly, past studies have shown sarcopenic individuals known to have increased levels of high sensitive C-reactive protein (CRP) and inflammatory cytokines IL-6 and TNF-α, compared to those without sarcopenia [18], and observational studies have shown these cytokines are associated with future muscle loss [19]. Two recent meta-analyses have been conflicting regarding TNF-α and IL-6, but both conclude elevated CRP is associated with sarcopenia [20,21]. The signalling pathways of these catabolic factors have all been shown to increase the expression or activity of the transcription factors Forkhead Box O (FOXO), including FOXO1 and/or FOXO3. The catabolic and anabolic pathways interact as the FOXO transcription factors can also be phosphorylated by Akt leading to exclusion of the FOXO transcription factors from the nucleus and thereby their inactivation [14].

Other key processes in regulating protein catabolism are oxidative stress, which is characterised by surplus reactive oxygen species (ROS), and energy balance where an energy deficit (measured by an increased in cytoplasmic adenosine monophosphate [AMP]) increases the activity of AMP-activated protein kinase (AMPK) [22]. Under physiological circumstances, ROS is important in the role of cell signalling and autophagy, but enhanced ROS production occurs in disease states, surgery and ageing [23]. Pathophysiological levels of ROS and activated AMPK both increase the activity of FOXO transcription factors thereby increasing the expression of MuRF and Atrogin. Skeletal muscle has an especially high metabolic rate, and is therefore particularly susceptible to damage [24]. Additionally, oxidative stress may also lead to chronic low grade inflammation [25].

MicroRNAs (miRNAs) are non-coding RNAs that are increasingly implicated in muscle wasting associated with disease states. Several different miRNAs have been found to interact with different pathways associated with muscle protein turnover. For example, miR-29b blocks IGF-1 and PI3K resulting in muscle atrophy[26]. Muscle biopsies in humans have shown miRNAs such as miR-542 and miR-424 to be over-expressed in those with intensive care unit-acquired weakness compared to controls whereas the expression of miR-181a, miR-1 and miR-131 were reduced [27-29]. Indeed, some disease states such as chronic obstructive pulmonary disease (COPD) have also been linked to higher levels of miR-424 and -542 suggesting underlying disease state may stimulate expression [27,28].

### Pathophysiology in surgery

#### Hormonal factors

Of the few studies examining the pathophysiology of surgery and muscle wasting, one prospectively compared different proteins in those who suffered muscle wasting against those who did not, following elective cardiac surgery. Myostatin levels unexpectedly fell in both groups following surgery and returned to normal by day 7. GDF-15 levels increased dramatically in both groups; levels returned to baseline at day 7 in the non-wasting group, but remained significantly elevated in the wasting group [29]. In the same study, IGF-1 fell in both the wasting and non-wasting group in the 2 days following surgery, but returned to normal in the latter group, while in the wasting group it remained suppressed. These findings were confirmed in a repeat study investigating the metabolic response on patients to aortic surgery [30]. In this study, pre-surgical levels of GDF-15 were higher in patients who went on to lose more than 10% of the cross-sectional area of the rectus femoris (wasting patients). The metabolomic analysis also showed that whilst the overall increase in cortisol was the same in wasting patients compared to non-wasting patients, cortisol metabolism differed such that the cortisol:cortisone ratio remained high in wasting patients compared to non-wasting patients.

Overall, there is a suggestion that imbalances in GDF-15 and IGF-1 contribute significantly to post-operative muscle wasting rather than changes in myostatin. The pattern of change in myostatin levels observed was consistent with another study, comparing those undergoing elective orthopaedic surgery to elective cardiac surgery in which myostatin levels also fell significantly by day 4, and began rising again by day 30, in both surgical groups, although outcomes of muscle wasting were not recorded. The reduction in myostatin was more pronounced in the orthopaedic group compared to cardiac surgery [31].

#### Inflammation

The trauma of surgery leads to an inflammatory response and this has been long touted as a key potential mechanism of muscle wasting after surgery [32]. While surgery has been shown to be associated with raised markers of inflammation, and, separately, inflammation itself is associated with muscle wasting, very few studies have looked at associations between markers of inflammation and muscle wasting after surgery. A small pilot study actually unexpectedly found a positive correlation between increased levels of CRP and bilateral anterior thigh thickness post-surgery (median day 7) [33]. One other study investigating the metabolic consequences of surgery showed that circulating levels of the chemokines C-C motif chemokine 23 (CCL23) and IL-8 were positively associated with the levels of amino acids in circulation whereas IL-5 was negatively associated with circulating amino acids following surgery [30]. The data were interpreted as showing that a range of inflammatory factors contribute to the loss of muscle mass following surgery.

#### Oxidative stress

Oxidative stress occurs in surgery through various means. Ischemia-reperfusion injury has been shown to induce this in different aspects of surgery, particularly transplantation, unclamping of the aorta (as in thoracic or abdominal aortic surgery), release of limb torniquet during orthopaedic surgery and reperfusion in coronary bypass surgery [34]. In a study comparing surrogates of oxidative stress following emergency surgery for hip fractures (“group A”) and elective hip replacements (“group B”), it was found both groups’ GSH:GSSH (reduced:oxidise gutathione) ratio decreased, indicating presence of oxidative stress [35]. However, group A’s decreased to a significantly greater extent than group B from the time of surgery up to day 15 post-operation. The antioxidant catalase was also significantly lower in group A preop, but increased significantly following admission. Conversely, group B’s catalase levels dropped, but remained higher than group A at all time points. It was concluded that emergency hip fracture surgery caused a greater extent of oxidative stress than elective hip replacements, and surgery stimulated a very active second line of oxidative defence (shown by increase in catalase) in hip fracture patients.

#### MicroRNAs

In a study of aortic surgery, those that went on to lose muscle expressed greater quantities of miRNAs from the miR-424/503 cluster (in particular miR-424, 542-3p and 5p) pre-operatively in their muscle biopsies [27,28]. Conversely, pre-surgical miR-422a was inversely associated with post-surgical muscle loss [36]. The expression of miR-424 and miR-542 are associated with disease severity as their expression in muscle is associated with lung function in patients with COPD and with cardiac function but not lung function in patients undergoing aortic surgery [28,37]. However, these miRNAs are better correlates with muscle loss and muscle function than classic markers of disease severity in both conditions. Furthermore, their targets suggest a direct role in the regulation of muscle protein homeostasis as both miR-424-5p and miR-542-3p target the protein synthetic machinery with miR-542-3p also promoting mitochondrial dysfunction and TGF-β signalling [27]. miR-422a which is negatively associated with muscle loss following surgery inhibits TGF- β signalling by targeting SMAD4 [36]. This suggests certain miRNAs may be able predict muscle wasting risk and the risk is likely to have already been inherently established prior to surgery [12].

#### Disuse

It has been well established that muscle disuse leads to reduced lean body mass and reduced strength, even in healthy individuals [38]. Disuse may exacerbate the risk of muscle wasting through impaired insulin sensitivity, inflammation, with pathways involving myostatin, Atrogin and MuRF1 shown to be involved [39,40]. Surgery confers a high risk of immobility that can be multifactorial - pain, need for non-weight bearing status after certain surgeries, and reduced functional state, from either underlying disease or the effects of surgery and anaesthesia itself, are all likely to contribute to this deleterious state.

#### Nutritional

**Figure 2.**
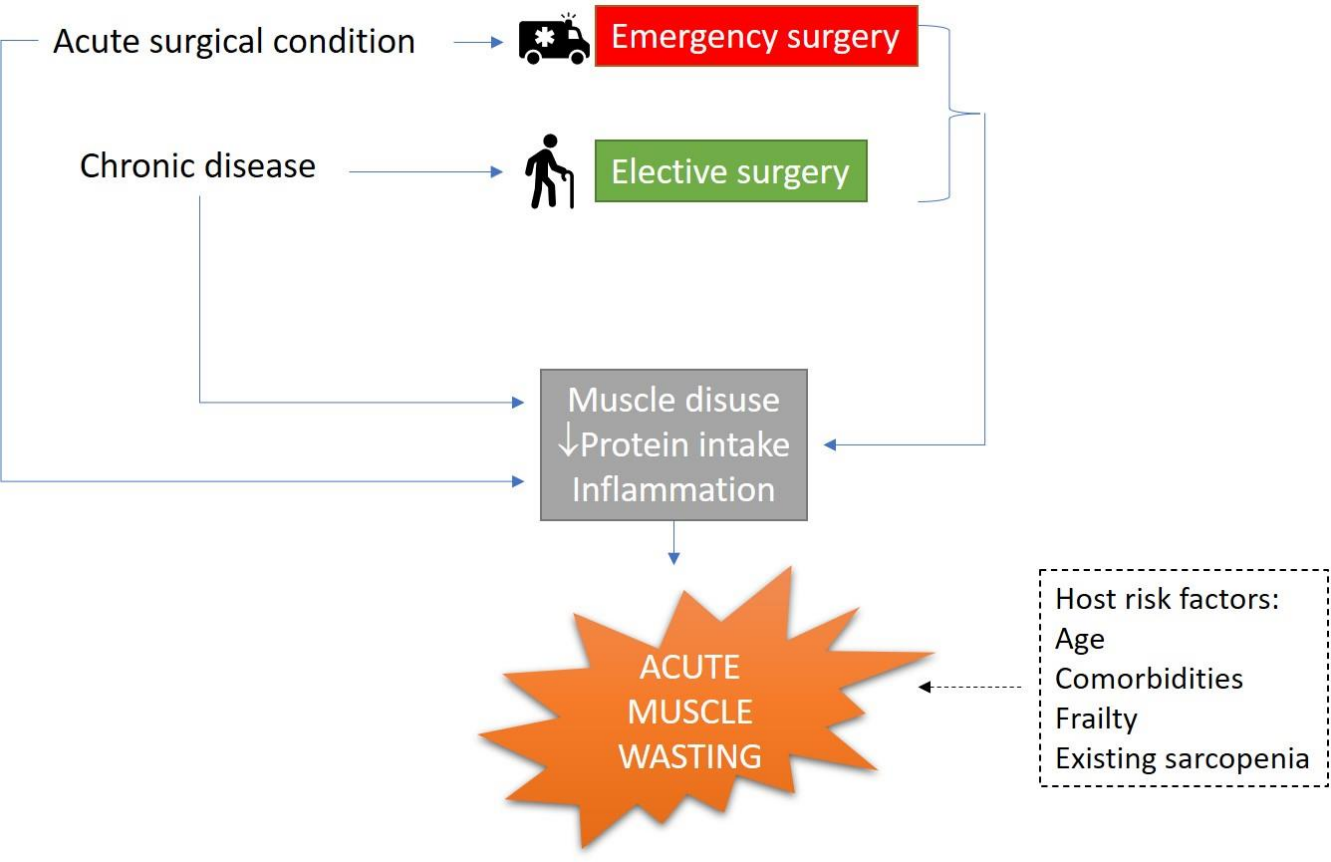
risk factors and causes of muscle wasting in the surgical patient.

As protein intake is an essential component of muscle maintenance, deficiencies from either reduced intake or malabsorptive states can directly cause muscle wasting [4]. This is highly relevant in the surgical patient. For example, those with an “acute abdomen” may be anorexic or vomiting for days prior to their emergency laparotomy. Additionally, most surgeries require a period of pre-operative fasting to reduce aspiration risk [41]. Furthermore, individuals undergoing certain surgeries, typically gastrointestinal, are recommended to continue fasting post-operatively for various reasons (including to reduce risk of vomiting and protect any surgical anastomoses), and even once feeding has recommenced, the build-up back to a normal diet is usually prolonged [42]. The interactions leading to sarcopenia in the individual undergoing surgery is summarised in Figure 2.

### Sarcopenia incidence and outcomes in surgery

#### Orthopaedic surgery

The prevalence of osteoarthritis rises with age, and subsequently the incidence of orthopaedic surgery, such as hip replacement, in older adults has also increased with an ever growing population [43,44]. The rate of sarcopenia was reported as high as 44% in patients undergoing a mix of elective and emergency orthopaedic surgery [45].

Numerous studies have examined muscle mass by CT cross sectional area (CSA), at various post-operative time points in lower limb surgery. In elective total hip replacement surgery, Kouw et al found CSA declined by 4.2% in the non-operated leg, during the inpatient stay of mean 5.6 days [46]. This is in keeping with previous experimental studies showing similar muscle loss from immobilisation alone [38,39]. It is worth noting that the decline of 4.2% in Kouw’s study is a mean change, and there will be variability amongst individuals. A standard deviation of ±4.9% is derived from the given standard error of mean of 1.1%, suggesting some may not lose any muscle, while others may lose a great deal (e.g., up to 9.1%). Also interesting is that at the point of hospital admission, the CSA of muscles on the operated side was lower to start with, when compared to the non-operated leg, suggesting underlying osteoarthritis may influence pre-operative muscle wasting. During hospitalisation, the thigh muscle CSA on the operated side increased by 8.0% owing to oedema. A randomised controlled trial by Suetta et al in 2004 compared standard rehabilitation against either resistance training or neuromuscular electrical stimulation in elective hip replacement surgery [47]. The natural history of muscle loss in this cohort was suggested by the loss of muscle CSA seen on the operated side 5 weeks post-surgery, which remained 9% below baseline at 12 weeks post-surgery, in the standard treatment arm. The resistance training group had an unaltered CSA at 5 weeks, which actually increased 12% at 12 weeks. Mak et al 2020 reviewed psoas muscle CSA at a mean interval of 4 months between surgery and imaging in those that underwent unilateral hip arthroplasty [48]. They discovered muscle area is reduced significantly in the implant side compared to the non-operated side. While there was no baseline imaging to compare muscle area size pre- and post-operatively, the researchers did find that increasing severity of osteoarthritis on the non-operated side, reduces this discrepancy of muscle area between the two sides. This further suggests that osteoarthritis has a large, long-standing impact on muscle atrophy. An observational study of serial CT scans two years apart, following total hip arthroplasty (THA) actually showed an overall increase in muscle size in the operated leg, suggesting that THA results in the recovery of these muscles, perhaps due to increased activity after the operation [49].

Muscle changes following emergency surgery for hip fractures has also been studied. Miller et al showed that following emergency surgery for hip fractures, there remained a significant reduction in thigh muscle CSA on the operated fractured leg, compared to the non-fractured leg, at 2 months [50]. Such changes may be prolonged to 3.5 years according to a different study [51]. Post-operative handgrip strength in those surgically treated for acute hip fractures predicted early ambulation, as well as post-operative complications [52].

An observational study revealed poor reported quality of life amongst those at risk of sarcopenia who were undergoing hip surgery using the SarQoL questionnaire. A high score (a sign of better quality of life) also strongly correlated with tibialis anterior muscle thickness [53].

#### Transplant surgery

Despite older adults being considered as higher risk for transplant surgery, solid organ transplantation has become more common in older age. Once the risk is overcome, not only can transplantation offer longevity, but also a marked improvement in the quality of the recipient’s remaining life [54]. However, sarcopenia is common in transplantation and even occurs at younger age. Aetiology can be multifactorial and likely to include presence of end-stage organ failure, underlying disease process and associated inflammation, and poor nutrition. In the case of kidney transplantation, length of duration on dialysis was also shown to increase the risk of developing sarcopenia [55].

Presence of sarcopenia preoperatively was associated with increased mortality in liver transplantation [56]. Jeon et al retrospectively reviewed pre-operative and 1-year post-operative abdominal CT scans of psoas muscles for sarcopenia, in those undergoing liver transplantation. They discovered an incidence of 15% new sarcopenia post-operatively, and these individuals were 10 times as likely to die, in comparison to those that remained non-sarcopenic [57]. A recent study has also shown that a greater deterioration in skeletal muscle index is associated with higher rates of graft failure and mortality in liver transplant recipients [58].

Poor outcomes are mirrored in kidney transplants, whereby presence of sarcopenia at the time of transplant has been associated with graft failure, post-operative complications and increased mortality [55]. New sarcopenia or muscle changes following renal transplantation remains an under-researched area, however.

#### Gastrointestinal surgery

The risk of gastrointestinal malignancy increases with age, and, as malignancy is also associated with increased rates of sarcopenia, it is not surprising to see a high rate of sarcopenia in those with GI malignancy who may be amenable to surgical intervention [59,60]. The presence of sarcopenia has been shown to be associated with higher mortality in patients with solid tumours in a meta-analysis of various tumours treated both surgically and non-surgery [61]. Specifically, in those undergoing surgery for colorectal cancer, this association with mortality also holds true [59].

Studies have utilised pre-operative staging CT scans and post-operative surveillance CT scans in those with colorectal cancer to assess changes in muscle mass following surgery. Those with significant muscle wasting have been consistently shown to have higher mortality rates than those without, in both younger age and older age groups [62,63]. Interestingly, Argillander et al demonstrated mortality was highest in the group with baseline sarcopenia and acute wasting, followed by baseline non-sarcopenia and acute wasting, which was higher still than baseline sarcopenia without acute wasting. [62]. This suggests acute wasting may be more important than baseline status. The variability in muscle wasting was also demonstrated here with an overall muscle change of 1.6% (i.e., muscle gain) with a standard deviation of ±5.7% of muscle mass.

The prevalence of sarcopenia 6 months after gastrectomy for gastric cancer rose from 2% (pre-operatively) to 20% in a group of over 70-year-olds, which seemed to plateau to 22% at 1 year [64]. Patients had significantly reduced oral intake at 6 months, which improved (but did not return to baseline) at 1 year. This level of anorexia is likely to be an important additional factor to major surgery itself, in contributing to sarcopenia.

#### Cardiac and vascular surgery

Ageing naturally increases the prevalence of conditions that need vascular intervention, including aortic aneurysm and peripheral artery disease. Less invasive endovascular procedures have allowed those that have been traditionally high risk for open surgery, to nowadays undergo prognostically and symptomatically important intervention. As such, the prevalence of sarcopenic patients being considered and even undergoing vascular procedures is likely to be high. In one study of all adults admitted onto a vascular ward who subsequently had a CT abdomen, 41% were found to have radiologically diagnosed sarcopenia [65].

Existing muscle wasting at the time of elective intervention for abdominal aortic aneurysm has been shown to be associated with 30-day mortality for both endovascular aortic aneurysm repair (EVAR) and open surgery [66].

Changes in muscle after vascular intervention are lacking. One study did look at psoas muscle area (PMA) changes following EVAR [67]. It showed an overall decrease in muscle area, which occurred the most during the first year following the procedure. The relative change in PMA from baseline was also an independent risk factor for mortality.

Okamura found in separate studies of older adults undergoing cardiac valve surgery and coronary artery bypass graft surgery (CABG), sarcopenia (defined by low psoas muscle area) independently conferred an increased risk of mortality [68]. Few studies have looked at acute muscle wasting following CABG. A small observational study showed an overall decrease in fat-free muscle mass (by DEXA), which correlated to blood cortisol: testosterone and younger age [69]. Variability in mass change occurred with some losing up to 6.3 kg in fat-free mass, while others even gained mass (up to 2.7 kg).

Different interventions of varying invasiveness and physiological stress can be offered for cardiovascular diseases. For example, transcatheter valvular implantation (TAVI) has become increasingly common for severe aortic stenosis and may be offered over the traditional open valve surgery in those considered “high surgical risk” [70]. Such decision making is now often influenced by the findings of novel predictive risk factors, such as sarcopenia and frailty, associated with invasive surgical intervention. This has led to less invasive procedures being offered to those with frailty, and even the avoidance of all intervention altogether in cases of extreme frailty [71].

### Treatment options

Interventions for muscle wasting and sarcopenia have centred around physical activity, resistance training and nutrition.

The International Clinical Practice Guidelines for Sarcopenia (ICFSR) recommends resistance-based training based on meta-analyses showing its potential for improving muscle strength, mass and performance, in community community-based participants with existing chronic sarcopenia [72]. The guidelines also recommend protein supplementation, although the strength of evidence was regarded low. However, in a landmark study, Iuliano et al. recently completed a cluster RCT of enhanced diet (of protein and calcium) versus usual diet [73]. While definitive sarcopenia was not measured, they did find that enhanced diet significantly prevented loss of appendicular lean mass (which can be considered a surrogate for sarcopenia), compared to control. Even more importantly, they found an overall decrease in their primary outcome of fragility fractures and secondary outcome of falls. Inhibition of ACE has also been suggested as a therapeutic approach to reduce sarcopenia. However, a recent two by two factorial RCT of leucine supplementation and ACE inhibition failed to identify significant increases in muscle mass or physical performance in either arm of the study [74].

In a meta-analysis of a surgical population looking at both prevention of sarcopenia and treatment of existing sarcopenia, exercise again was shown to be the most effective intervention for outcomes in muscle mass, strength, performance and length of hospital stay [75]. Exercise that was provided early post-operatively was considered more beneficial than pre-operative or late post-operative. No evidence was found for dietary interventions. However, two randomised controlled trials completed following this study supported the use of nutritional interventions, in gastric surgery [76] and colorectal surgery [77]. Additionally, an RCT by Burden et al in 2017 demonstrated in the group of weight-losing colorectal cancer patients undergoing surgery, a period of pre-operative oral nutritional supplements was associated with reduced post-operative infections (an adverse outcome often linked to sarcopenia)[78]. In this study, the mean handgrip strength was 25 kg and thus likely represented a group of sarcopenic participants. This also highlights how interventions must be trialled and delivered in the right cohort of patients that are likely to derive the benefit.

The type of surgery may also be relevant. In an RCT comparing intervention of nutritional supplementation to control in those who have had emergency surgery for hip fractures, the intervention was not associated with any benefit in outcomes of handgrip strength or appendicular lean mass [79]. There is interest in pharmacological treatments that interfere with the pathway leading to sarcopenia. Growth hormone injections prevented loss of lean body mass, which occurred in the placebo group, in one RCT of surgical hip fracture participants [80]. In elective surgery, GH was found to ameliorate loss of muscle CSA that was found in the placebo group, although there was a 3-fold increase in symptoms or signs of fluid retention in the treatment group [81]. Other novel therapeutics include myostatin antibodies which have shown to improve lean body mass [82]. However, as myostatin seems to decrease following surgery, trials of these antibodies in surgical cohorts may have very limited benefit; additionally, the intervention was also associated with adverse injection site reactions.

### Areas for future research

Currently the definition of acute sarcopenia has been denoted as new sarcopenia arising at an arbitrary 6 months. It is unknown whether this is the optimal timing that captures clinically significant new diagnoses of sarcopenia. Future studies which serially monitor muscle strength and size following an acute event will help understand this more accurately. We also do not know whether establishing incident sarcopenia by a single cut-off of strength measurement is any more important than by measuring *changes* in muscle strength or size. Just as acute kidney injury uses changes in creatinine to define its acuteness over chronicity, it may be that a definition for acute sarcopenia should incorporate such loss of muscle strength or size to be relevant. For example, those with existing definite sarcopenia who then go onto lose further muscle strength following surgery would not be captured by the current definition of acute sarcopenia.

Studying those who lose muscle or become sarcopenic has important implications. It may be possible to predict such patients through biomarkers. By identifying this group, future clinical trials of intervention could potentially target those that have the most to lose and potentially the most to gain. Data showing that pre-surgical phenotype differs between those who lose significant muscle mass and those who do not suggests that sarcopenia following surgery may be an ideal place to identify such biomarkers [27,28,36].

### Conclusion

Sarcopenia is common in surgical patients pre-operatively and its presence is associated with increased adverse outcomes, including infection, post-operative complications, and death, across all surgical specialties. Whilst the landmark EWGSOP2 definition has shone light on sarcopenia and allowed a diagnosis to be established definitively, there remains a need for further research particularly in surgical patients, focussing on deterioration in muscle strength/mass and incidence of new acute sarcopenia post-operatively.

The pathophysiology of sarcopenia following surgery is not fully understood. There is evidence that GDF-15 and IGF-1 play key roles, along with an inflammatory state most consistently signalled by elevated CRP. Elevated baselines of certain miRNAs have also been shown to predict muscle wasting in cardiac surgery, but this is yet to be replicated in other forms of surgery. We have also highlighted how different types of surgery may invoke particular pathways to greater levels than other types. Understanding pathophysiology may pave the way for future therapeutic targets which also need exploring through high quality trials.
